# Mmp17b Is Essential for Proper Neural Crest Cell Migration *In Vivo*

**DOI:** 10.1371/journal.pone.0076484

**Published:** 2013-10-01

**Authors:** Noah R. Leigh, Marcus-Oliver Schupp, Keguo Li, Vakeel Padmanabhan, Adam Gastonguay, Ling Wang, Chang Z. Chun, George A. Wilkinson, Ramani Ramchandran

**Affiliations:** 1 Department of Pediatrics, Developmental Vascular Biology Program, Children’s Research Institute (CRI), Medical College of Wisconsin, Milwaukee, Wisconsin, United States of America; 2 Department of Medicine, Division of Nephrology, Hypertension and Renal Transplantation, University of Florida, Gainesville, Florida, United States of America; University of Colorado, Boulder, United States of America

## Abstract

The extracellular matrix plays a critical role in neural crest (NC) cell migration. In this study, we characterize the contribution of the novel GPI-linked matrix metalloproteinase (MMP) zebrafish *mmp17b. Mmp17b* is expressed post-gastrulation in the developing NC. Morpholino inactivation of *mmp17b* function, or chemical inhibition of MMP activity results in aberrant NC cell migration with minimal change in NC proliferation or apoptosis. Intriguingly, a GPI anchored protein with metalloproteinase inhibitor properties, Reversion-inducing-Cysteine-rich protein with Kazal motifs (RECK), which has previously been implicated in NC development, is expressed in close apposition to NC cells expressing *mmp17b*, raising the possibility that these two gene products interact. Consistent with this possibility, embryos silenced for *mmp17b* show defective development of the dorsal root ganglia (DRG), a crest-derived structure affected in RECK mutant fish *sensory deprived* (*sdp*). Taken together, this study has identified the first pair of MMP, and their putative MMP inhibitor RECK that functions together in NC cell migration.

## Introduction

The neural crest (NC) is a transient multipotential progenitor cell population arising from the dorsal folds of the neural tube [1,2]. NC cells undergo epithelial-to-mesenchymal (EMT) transition, and begin to migrate throughout the developing body, giving rise to the craniofacial skeleton, melanocytes and sympathetic and sensory ganglia [3,4]. Tissues surrounding the neural tube produce both positive and negative cues that guide NC cells along defined paths [5]. Trunk NC cell migration is guided by signals emerging from adjacent somites [6]. Once in the trunk, NC cell migrate either ventromedially or dorsolaterally, which eventually predicts their fate [5], however trunk NC cell are specified before reaching their final location [3]. NC cells migrating via the ventromedial route without invading the sclerotome (somite) become neurons and glia of the sympathetic ganglia, and adrenal chromaffin cells [1]. NC cells taking the ventromedial route invade and remain within the sclerotome to form Schwann cells, sensory neurons and glia of the dorsal root ganglia (DRG). NC cells that take a dorsolateral route in between the dorsal ectoderm and the dermamyotome differentiate into melanocytes [5]. In terms of molecular cues, trunk NC cells that migrate via the ventromedial route enter the somite via attraction cues from CXCR4/CXCL12 signaling molecules [7,8]. These NC cells are confined to the rostral sclerotome by Neuropilin2/Semphorin 3F repulsion molecules working in concert with Eph/ephrin signaling, F-spondin and proteoglycans that reinforce this migration route [8]. As with most cell migration processes, the extracellular matrix surrounding tissues play a critical role in directing NC cells to their final destination [9]. Matrix metalloprotease (MMPs) enzymes are responsible for degradation of extracellular matrix, and facilitate the migration and invasion of NC cells [10,11]. Recently, a group of molecules has been identified that control MMPs activity via direct inhibition. These molecules also contribute to the migratory routes taken by motile cells. For example, a zebrafish genetic mutant, *sensory deprived* (*sdp*) [12] carries a mutation in a glycophosphatidyl-inositol (GPI) anchored metalloprotease inhibitor protein Reversion-inducing-Cysteine-rich protein with Kazal motifs (RECK). *Sdp* mutant zebrafish show NC cell migration defects leading to defective DRG formation [12]. Despite these accepted concepts, the identification of specific MMPs involved in NC cell migration is still in its infancy. In this study, we extend the role of MMPs to NC cell development by identifying and characterizing the function of a zebrafish ortholog of MMP17 in embryonic NC migration.

## Results

### Biochemical characterization of the zebrafish *mmp17b* gene

**Figure 1:**
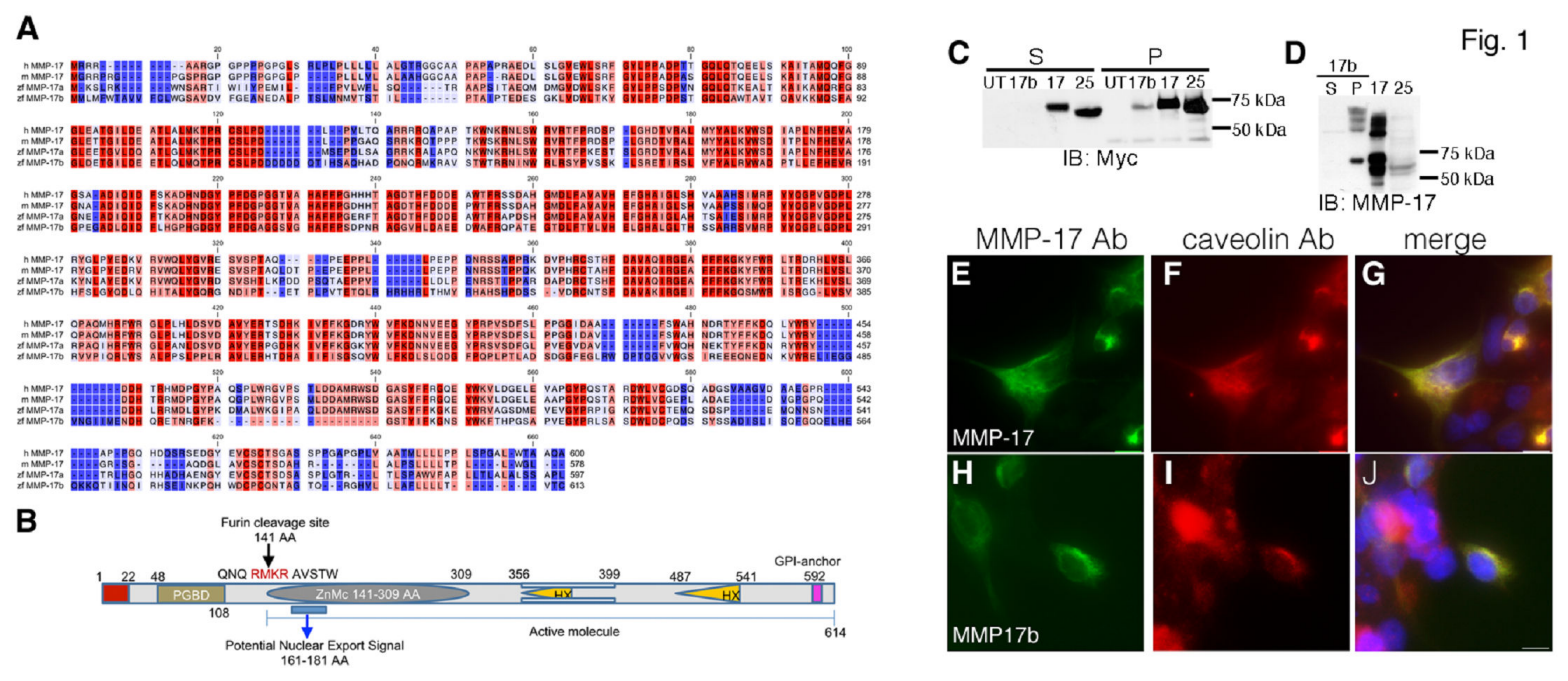
Bioinformatic and biochemical analysis of Mmp17b. Panel A depicts amino acid alignment of human and mouse MMP17 and zebrafish Mmp17a and Mmp17b. Red color indicates conserved amino acids and blue color indicates less conserved regions. B is a cartoon of Mmp17b protein. The predicted domains include a zinc catalytic domain, hemopexin-like domains, and a GPI-anchor. C and D are myc and MMP17 western blots of HEK293T cell lysates respectively. Supernatant (S) and pellet (P) fractions were generated as described in Methods S1. Mmp17b protein is observed only in the P fraction. MMP17 and MMP25 proteins are more robustly expressed, and were observed in both S and P fractions. D depicts bands at the proper size for Mmp17b and MMP17 with some cross reactivity to MMP25. Bands of higher molecular weight are also observed. UT = Untransfected, S = supernatant, P = pellet, + = positive control, E = empty vector control. E-J are myc tagged *MMP17* and myc-HIS tagged *MMP17b* cDNAs transiently expressed in HEK293T cells. The enriched metalloproteinase fusion proteins were detected using human specific MMP17 antibody (E & H; shown in green) and a caveolin mAB antibody (F & I; shown in red). The overlay images (G & J) show co-localization of caveolin with MMPs in positively transfected cells. Image micrograph depicting nucleus stained with DAPI not shown. Scale bars are 10 micron.

We performed a search for transcripts in the zebrafish information network (ZFIN) using expression pattern keywords such as vessels (Methods S1). These searches identified an expressed sequence tag (EST) sb:eu434 showing a segmental expression pattern along the embryonic trunk reminiscent of the intersomitic vasculature. A BLAST search revealed that the sb:eu434 EST sequence showed 56% sequence homology with matrix metalloprotease17 (MMP17; MT4-MMP) (Figure S1A). As part of a whole genome effort to characterize MMPs, a previous study has identified using bioinformatics a *MMP17* ortholog (*mmp17a*) in zebrafish [13]. We therefore renamed the EST as *mmp17b*. The *mmp17b* gene consists of 11 exons, predicted to encode a 613 AA protein. Analysis of the predicted Mmp17b protein product using multiple web based programs [14,15] identified a zinc-dependent metalloprotease domain (AA141-309) (Figure S1C), two hemopexin-like (HX) domains (Figure S1C) (AA 356-399 and 487-541) and GPI anchor site (AA 592). To confirm the bioinformatic prediction, we cloned the full-length zebrafish *mmp17b* cDNA into pcDNA3.1 with a myc tag (Figure 1C, Methods S1) to determine expression via western blot using myc (Figure 1C) and MMP17 antibodies (Figure 1D). We also included cDNAs for two of the identified human GPI-linked MMPs, MMP17 and MMP25 [16] as controls. We transfected 293T cells with myc expression constructs, and generated soluble (S) supernatant and insoluble pellet (P) fractions, that were probed with myc and MMP17 antibodies. Western blots with myc antibody (Methods S1) showed a ~70 kDa band in Mmp17b pellet lysate lanes (Figure 1C) similar to human MMP17 (~73 kDa) and MMP25 (~68 kDa). The S fractions also showed bands at the respective sizes although the intensity was quite weak. Interestingly, the human MMP17 antibody cross-reacted with the zebrafish Mmp17b protein in 293T lysate (Figure 1D), which was again observed predominantly in the P fraction. Since GPI-anchored proteins are often localized in caveolin-rich membrane fractions [17], we performed immunofluorescence (IF) with human MMP17 and caveolin antibodies on 293T cells transfected with human MMP17 (Figure 1E-G) and zebrafish Mmp17b (Figure 1H-J) cDNAs (Methods S1). Both human MMP17 (Figure 1G) and Mmp17b proteins (Figure 1J) are co-localized with caveolin in 293T cells. Taken together, these results support the prediction based on sequence homology that the *mmp17b* gene is a GPI-anchored MMP, and is a putative ortholog of the GPI-family member MMP17.

### Expression analysis of *mmp17b* across embryonic development

**Figure 2:**
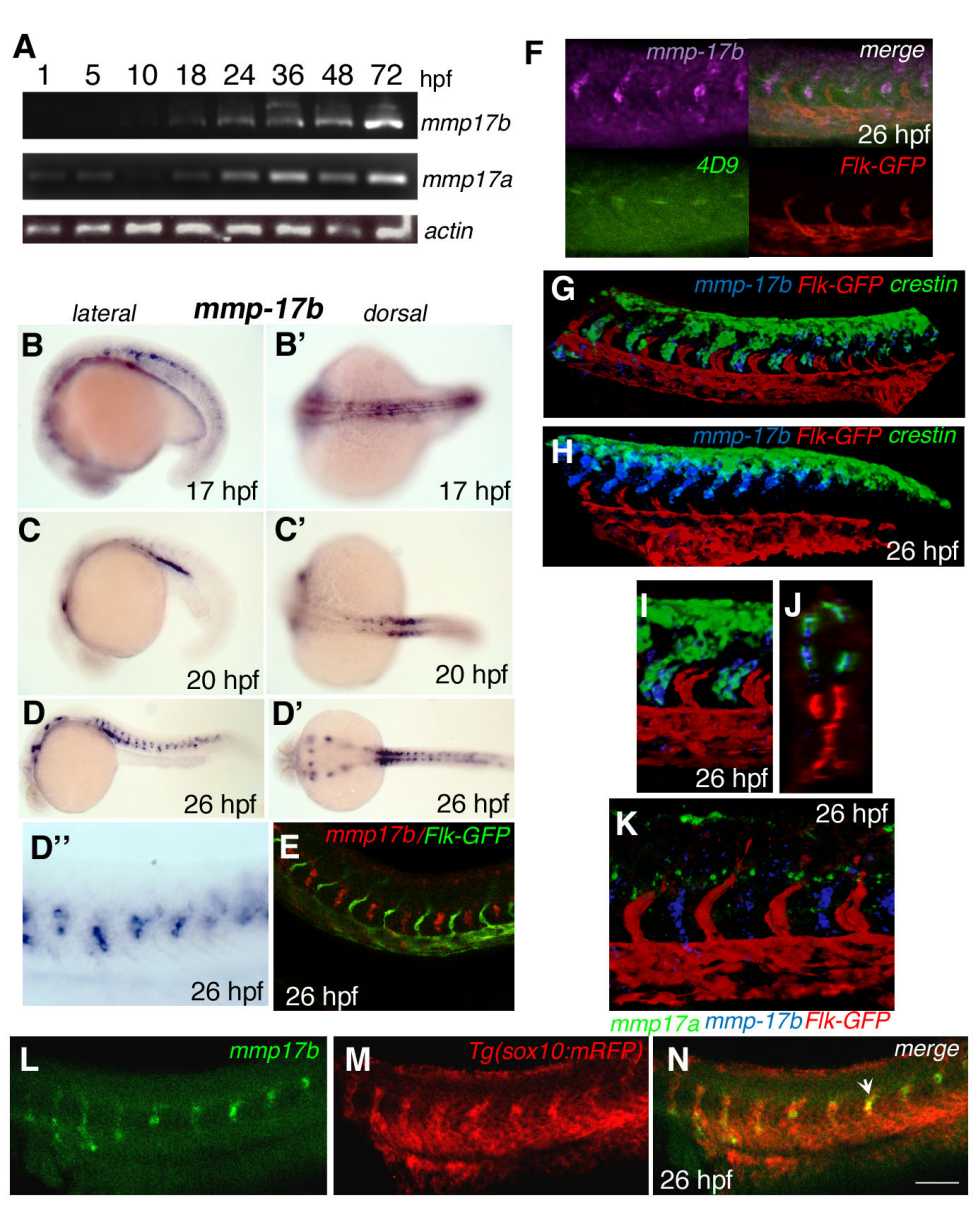
Spatial and temporal characterization of *mmp17b* expression. A is RT-PCR showing temporal expression of *mmp17b* and *mmp17a* compared to β-actin. *Mmp17b* expression commences at around 18 hpf while *mmp17a* is expressed as early as 5 hpf. B-E are whole mount in situ hybridization (WISH) panels of *mmp17b* at different embryonic stages (B and B’, 17 hpf; C and C’, 20 hpf; D and D’, 26 hpf). Earlier in development *mmp17b* is expressed more dorsally and then moves more posterior and ventral as development continues. Panel E shows *mmp17b* fluorescent in situ hybridization (FISH) in red and immunofluorescence (IF) for Flk-GFP in a 26 hpf embryo, which illustrates lack of *mmp17b* expression in the vasculature. Panels F, G-H, I-J and K are three color WISH staining performed as described in materials and methods with different probes as indicated in each panel. In panel F, *mmp17b* (purple), lower left is 4D9 staining (green) the muscle pioneer cells, lower right is Flk-GFP marking (red) the endothelial cells, and upper right is a merge. In this panel you can observe that 4D9 staining is located in the same region as *mmp17b*. G-H panel shows three color ISH image of 26 hpf zebrafish trunk and plexus with *mmp17b* (blue), Flk-GFP (red), and *crestin* (green). In the trunk image, *crestin* and *mmp17b* are overlapping in expression suggesting co-expression of these two genes in the same cell. The expression of both *crestin* and *mmp17b* are more dorsal in posterior regions of the embryo. Panels I-J represent high-powered image of the panel G. J is an optical section of panel G. Panel K is a three color image of 26 hpf zebrafish trunk with *mmp17a* (green), *mmp17b* (blue), and Flk-GFP (red). This image illustrates that *mmp17a* and *mmp17b* are expressed in different regions of the developing embryo. B-D are lateral views, B’-D’ are dorsal views. D” is a close-up of the panel D. Panels L-N are single plane confocal images of a 26 hpf embryo stained for *mmp17b* using FISH (panel L, green), and immunostaining for sox10 cells labelled with RFP (panel M, red). Panel N is the merged images showing co-localization of *mmp17b* and sox10 as indicated by yellow color (arrowhead).

To investigate the temporal expression pattern of *mmp17b* during embryonic development, we performed RT-PCR on total RNA isolated from stage-specific wild type strain TübingenAB (TuAB) zebrafish embryos using gene-specific primer sets for *mmp17b, mmp17a* and *actin* (Table 1 and Figure 2A). *Mmp17a*, but not *mmp17b* was observed between 1-10 hpf which means *mmp17a* is a maternally derived transcript. Expression of *mmp17b* is first detected at 18 hpf and continues through 72 hpf with most robust expression observed between 24-72 hpf. To determine the temporal and spatial expression pattern for *mmp17b*, whole mount in situ hybridization (WISH) was carried out on 17, 20 and 26 hpf embryos. At 17 hpf, discontinuous *mmp17b* ISH staining is visible in the neural tube (NT) region (Figure 2B). At 20 hpf, *mmp17b* transcript is observed in the ventral mesoderm tissue - intermediate cell mass (ICM) region adjacent to the yolk extension (Figure 2C). At 26 hpf (Figure 2D, arrows), *mmp17b* ^+^ cells appear throughout the middle of the trunk region, which on high power are observed adjacent to the somite boundaries (Figure 2D’’). To elucidate the cell types that express *mmp17b* in the trunk, we performed double staining by combined fluorescent in situ hybridization (FISH) for *mmp17b* and immunostaining for Flk-GFP (vascular) (Figure 2E) or znp-1 (cell body – motor neuron)/zn-1 (axon - motor neuron) (Figure S2A) or 4D9 (muscle pioneer cell) (Figure 2F) or immunostaining for RFP using a *Tg*(*sox10*(*7.2*)*:mrfp*) line or ISH for *crestin* (NC) (Figure 2G-J) marker. *Mmp17b* messenger RNA is not detected in *flk* positive ISVs (Figure 2E) but is readily detected within the intervening somites. *Mmp17b* expression appears to co-localize with znp-1/zn-1^+^ motor neurons (Figure S2A), and to some extent with 4D9^+^ muscle pioneer cells (Figure 2F). *Mmp17b* clearly co-localizes with *crestin* positive (*crestin* ^*+*^) NC cells in the trunk at 26 hpf (Figure 2G & 2J), and interestingly this co-localization is observed in the anterior somites (Figure 2G & 2I) where *mmp17b* ^+^ cells have migrated more ventrally than *crestin* ^*+*^ cells. Our staining procedure includes a tyramide signal amplification process. Tyramide is a precipitate that prevents the combination of colors (e.g., red and green equals yellow) that is normally used to indicate co-localization. Since one precipitate covers the other, we looked for cells that appeared to have both colors represented on them to indicate co-localization. To confirm this observation, we also conducted FISH for *mmp17b* in *Tg*(*sox10*(*7.2*)*:mrfp*) [18] embryos, and we also observed co-localization in *sox10* ^*+*^ NC cells at the same stage (Figure 2N, arrowhead). In parallel experiments, *mmp17a* expression was detected across the dorsal side of the embryo, more dorsally than seen for *mmp17b* (Figure 2K). Taken together, we observe *mmp17b* expression post gastrulation in tissues that undergo morphogenesis and patterning, and in particular in *crestin* ^*+*^ NC cells.

**Table 1:** Primer and Morpholino Sequences used in this study.

**Primer and Morpholino Sequences**	
**Primers**	
*mmp17a* probe (PCR)	Fwd: CCTGGAGGAAACAGGAGTGT
	Rev: caccgaaattaaccctcactaaagggAGGACTCAATGGCAGAGGTGT
*crestin* probe (PCR)	Fwd: ACATGAGGTCAGACGACTAACGC
	Rev: caccgaaattaaccctcactaaagggAGATTCCACGGTCTCTCCCGGTGAC
*reck* probe (PCR)	Fwd: ATGAGCGGGTGTCTCCAGATCC
	Rev: caccgaaattaaccctcactaaagggAGAAACACTGCCAGAGCGGGTCC
*mmp17b* pCS2+ cloning	Fwd: agc*tctaga*AGCAGAGGGAGGATCAGAGAAGC
	Rev: agc*tctaga*TGGGGTCAACTGTCCCTTTATTAACAAG
*timp2a* probe	Fwd: ATGAAGAGCGTCAGGAGCTGTATT
	Rev: CACCGAaattaaccctcactaaagggagaTAAGGGTCTTCCACATCCAGAAACtct
*timp2b* probe	Fwd: ATGAGTATGTCTCGGTCAGTTCCTct
	Rev: CACCGAaattaaccctcactaaagggagaATGTATTCTCATGGTTCCTCGATGTCCA
*mmp17b* forward Cloning	Fwd: TTT*ggtacc*GAGCCACCATGATGCTGATGTTTTGGACTGCAG
	Rev: TTT*gcggccgc*ACAAGTGACAGTGAGCAGAAGCAG
*mmp17b* Targeting (PCR)	Fwd: GTGTTTGCTCTCGGGATGAT
	Rev: CAACAGATCCGCCTGCTC
β-actin (PCR)	Fwd: CATCAGCATGGCTTCTGCTCTGTATGG
	Rev: GACTTGTCAGTGTACAGAGACACCCTG
**Morpholino**	
*mmp17b* MO1	GACTGGGAAAATAAACATACCTAAT
*mmp17b* MO2	GCTAAAATAATCAGCCGAACCTCCA
Control MO	CCTCTTACCTCAGTTACAATTTATA

Small letter sequences indicate T3 RNA polymerase-binding site with base pair overhangs. Italic small letter sequences indicate restriction enzyme sites with base pair overhang to facilitate restriction enzyme digestion.

### *Mmp17b* loss of function (LOF) embryos show defects in trunk NC cell patterning

**Figure 3:**
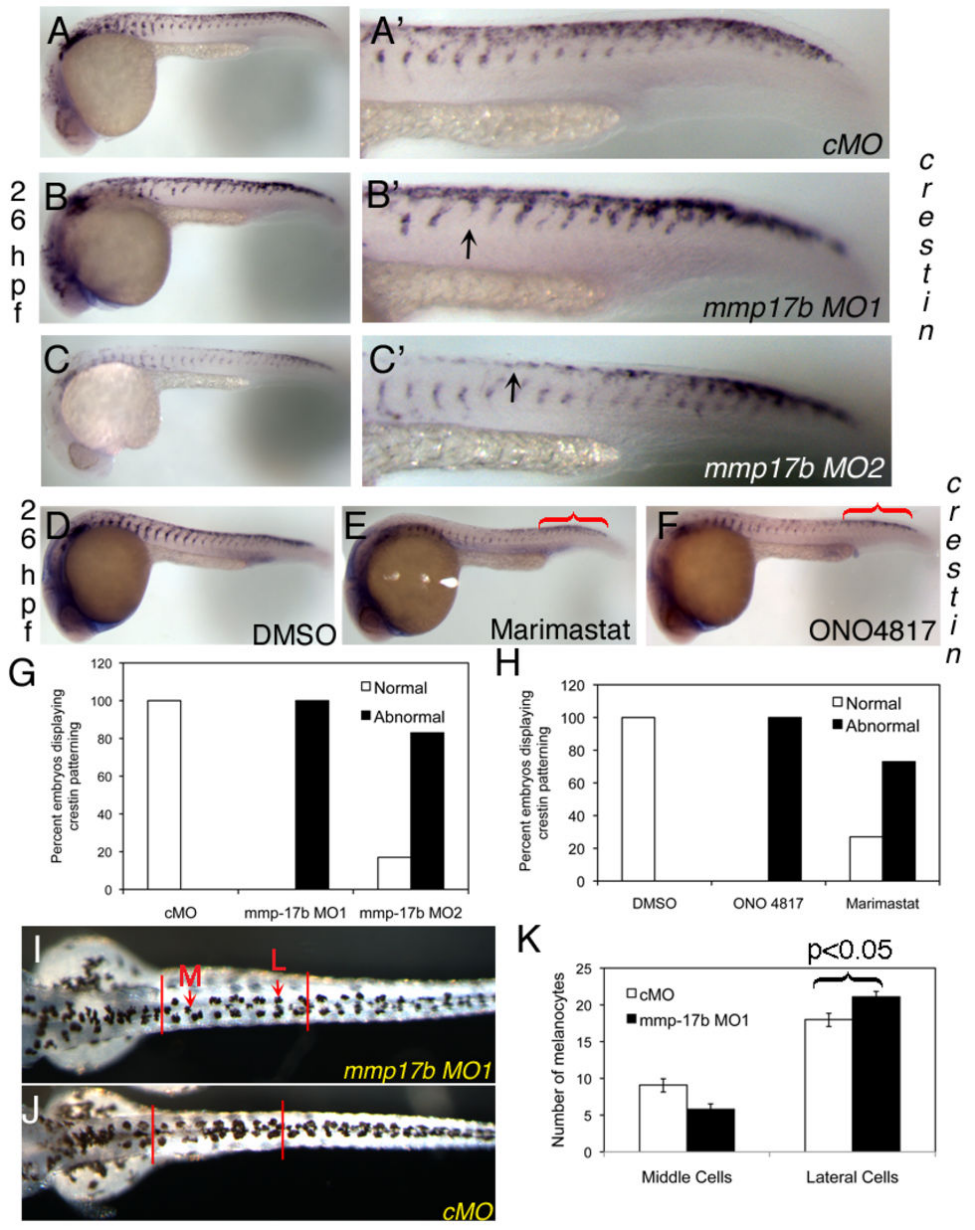
Mmp17b is involved in neural crest patterning. A-C and D-F are WISH staining for *crestin* in control MO (cMO) (A), MO1 (B), and MO2 (C) injected 26 hpf or DMSO (D), Marimastat (E) and ONO-4817 (F) treated 26 hpf embryos. A’-C’ are high powered images of the trunk regions of A-C. Arrowheads indicate *crestin* ^*+*^ cells misplaced in the trunk. There is a mis-patterning of *crestin* in the trunk of the MO1 and MO2 injected embryos compared to control. There is also an accumulation of *crestin* ^+^ cells in the posterior of the embryo (white bracket) compared to control. This is quantitated in panel G. N=25 for cMO; n=19 for MO1; n=18 for MO2. In panels D-F, WISH staining for *crestin* in MMP inhibitor treated 26 hpf embryos shows mis-patterning similar to *mmp17b* KD embryos (A-C). An accumulation of *crestin* ^+^ cells in the posterior of the MMP inhibitor treated embryos is also observed (red brackets). This is quantitated in panel H. N=12 for DMSO; n=8 for ONO 4817; n=10 for Marimastat. I-K shows melanocyte quantitation done on 72 hpf fish. Dorsal images were taken of 72 hpf fish injected with either control MO (J) or *mmp17b* MO1 (I). The number of medial (M, red arrow) and lateral (L, red arrow) melanocytes is counted between the two vertical bars illustrated in panels I and J for 10 fish in each category. The results were quantitated in panel K. The medial cells were not statistically different but the lateral cells were at a p-value of less than 0.05.

To assess function of *mmp17b*, we designed two separate splice morpholinos (MOs) that target the exon-intron junction of exons 3 and 4 of *mmp17b* (Figure S2B), and validated the MOs using RT-PCR with exon-specific primers spanning the targeted junctions (Figure S2B). Injection of 7.5 ng of MO1 resulted in expression of an abnormal higher molecular weight gene product (Figure S2C, lane MO1) because of intron retention, which resulted in an in-frame stop codon. Injection of MO2 at 12.5 ng dosage resulted in multiple aberrant RT-PCR products (Figure S2C, white asterisk). We performed WISH at 14 hpf, a stage prior to initial detection of *mmp17b* transcript (Figure 2A) for *myoD* (somites) [19], and for *etv-2* (early endothelial hemato-vascular precursor cells) [20] in control and *mmp17b* injected morphants (Figure S2D, compare CMO vs. *mmp17b* MO1). We observed no alteration in these structures implying that the loss of *mmp17b* does not affect the development of somites or endothelial precursors during embryonic development. Based on the co-expression pattern of *mmp17b* and *crestin* (Figure 2G), we investigated *crestin* ^*+*^ NC cells in *mmp17b* morphants, and observed that both *mmp17b* MO1 (Figure 3B & B’) and MO2 (Figure 3C & C’) -injected embryos showed defective *crestin* patterning in the trunk region (arrowheads in Figures 3B’ & 3C’) compared to control MO (cMO)-injected embryos (Figure 3A). This abnormal *crestin* patterning was quantified (Methods S1), and greater than 80% of embryos displayed *crestin* patterning defects in *mmp17b* morphants (Figure 3G). The crestin patterning defect is defined as the irregular spacing of *crestin* ^*+*^ cells in the trunk presumably due to altered migration. Complementary gain of function with mRNA injection and rescue experiments were attempted by co-injecting human *MMP17* or zebrafish *mmp17b* mRNA along with *mmp17b* MO. Unfortunately both gain of function (GOF) and rescue experiments caused embryonic death, even at low doses implying stress-like responses in early embryos to presence of MMP RNAs at stages when they are not expressed (before 18 hpf). Our *mmp17b* LOF study indicates a critical role for *mmp17b* in *crestin* patterning in the trunk of developing zebrafish embryos.

### Small molecule inhibition of MMPs results in a neural crest phenotype similar to *mmp17b* morphants

We utilized a complementary chemical biology approach to address the function of *mmp17b* in embryonic development, an approach extensively used in zebrafish to perturb developmental processes [21]. We investigated several broad-spectrum MMP inhibitors [22,23] and selected Marimastat, a collagen peptidomimetic, and ONO-4817, a synthetic hydroxamic acid- based non-peptidomimetic (Figure S3A-B), for further experiments. We incubated dechorionated or chorion punctured Tg(*kdrl*:EGFP) embryos in 0.5 mM of each compound from 10 to 26 hpf followed by fixation in 4% PFA. *Crestin* WISH was performed on the MMP inhibitor-treated as well as DMSO-treated control embryos. The embryos treated with both MMP inhibitors (Figure 3E & 3F) showed similar alterations of number and positioning of *crestin* ^*+*^ cells to that seen with *mmp17b* MO-injected morphants (Figure 3B & 3C). There are fewer *crestin* ^*+*^ cells in MMP-inhibitor-treated embryos compared to the DMSO-treated embryos, and the *crestin* ^*+*^ cells that are present in MMP-inhibitor-treated embryos (Figure 3E & 3F, compare red bracket) are mislocalized when compared to controls. Quantification of the defects shows greater than 80% of embryos have defective *crestin* patterning in both MMP inhibitor-treated embryos (Figure 3H).

To determine whether *crestin* ^*+*^ cells in *mmp17b* morphants were undergoing apoptosis, we performed a terminal deoxynucleotidyl transferase dUTP end labeling (TUNEL) assay as described in Methods S1. As a positive control for our assay, we treated embryos with 6 mM hydrogen peroxide, which results in generalized apoptosis, and was detected in our staining method (Figure S3D). However, in uninjected (Figure S3C, UI) or *mmp17b* MO1-injected (Figure S3E) embryos, we noticed no difference in TUNEL staining. We next investigated the possibility of altered cell proliferation in *mmp17b* morphants by performing phospho-histone3 (pH3) staining (Figure S3F-H, Methods S1). Although we noticed a slight increase in the overall pH3^+^ cells in the trunk of the *mmp17b* KD embryos (Figure S3H), these results were not statistically significant. Taken together, the LOF-MO and the MMP inhibitor results support the hypothesis that *mmp17b* affects trunk NC cell development in embryonic zebrafish.

### *Mmp17b* is involved in trunk NC cell migration

In both *mmp17b* morphants (Figure 3B & 3C, compare white bracket area) and MMP-inhibitor-treated (Figure 3E & 3F, compare red bracket area) embryos, the uniform distribution of *crestin* ^*+*^ cells in the dorsal region of the embryo observed in control-MO (Figure 3A) or DMSO-treated (Figure 3D) fish was disturbed. In morphant or inhibitor treated fish, *crestin* ^*+*^ cells are detected more posteriorly along the dorsal axis compared to controls, suggesting a change in migration pattern. Trunk NC cells that migrate and differentiate along dorsolateral routes become melanocytes [5]. Therefore, we investigated melanocyte spatial distribution in embryos injected with cMO (Figure 3I) or *mmp17b* MO1 (Figure 3J) at 48 hpf. Embryos injected with *mmp17b* MO1 showed an increase in melanocyte cells that were located on either side of the midline (lateral melanocytes) (Figure 3K, lateral cells, p<0.05) compared to control-MO while no difference (Figure 3K, medial cells, p>0.05) was noted in cells located at the midline (medial melanocytes). However, the total number of melanocytes between the control-MO and the *mmp17b*-MO1-injected fish were roughly equal. These observations suggest that melanocyte differentiation from *crestin* ^*+*^ cells is unaffected but that their final destination in the trunk has changed. Together, these results suggest that loss of *mmp17b* results in trunk NC cell migration defects in the developing zebrafish embryo.

### Mmp17b and its putative inhibitor (Reck) are proximally expressed in the trunk, interact biochemically, and are involved in DRG formation

**Figure 4:**
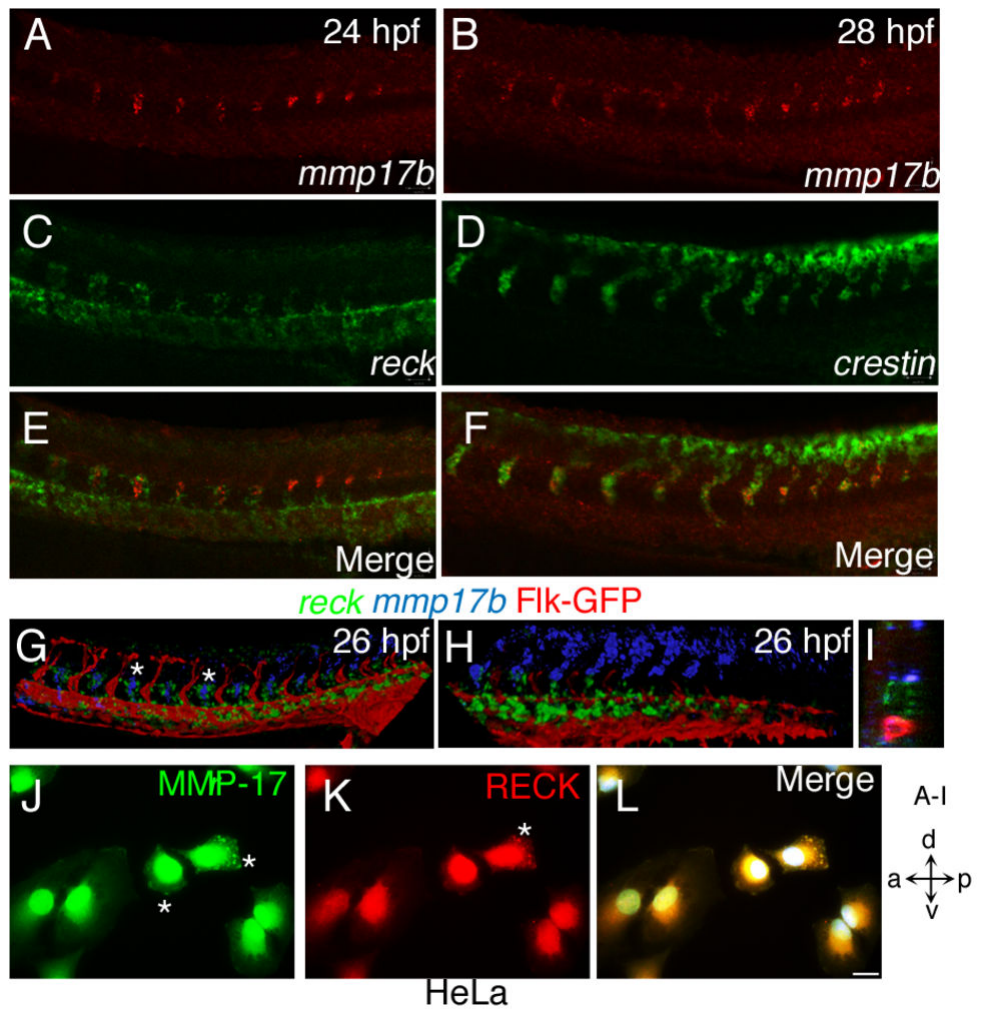
Mmp17b and Reck are involved in DRG formation. A, C and E are 26 hpf WISH embryos for *mmp17b* (A) in red and *reck* (C) in green. Panel E is a merge of the two images. *Reck* and *mmp17b* are expressed near each other in the trunk at this time point. B, D, and F are 26 hpf WISH embryos for *mmp17b* (B) in red and *crestin* (D) in green. Panel F is a merge of the two images. *Mmp17b* and *crestin* are co-expressed in the trunk at this time point. G, H, and I are confocal image of three color staining of *mmp17b* (blue), *reck* (green), and Flk-GFP (red) in the trunk (G) and plexus region (H) of 26 hpf embryos. Asterisks show that *mmp17b* and *reck* are expressed next to each other but not in the same cell. Optical section of panel G shown in panel I shows the proximity of *mmp17b* and *reck* expression. Panels J-L are cultured HeLa cells on glass coverslips double stained with MMP17 (J, Green) and RECK (K, Red) antibodies. The overlay of image (L) shows both MMP17 and RECK localizes within the cytoplasm in a punctate manner and near the leading edges of the cell indicated by asterisk (*). Image panel stained with DAPI not shown. Scale bar 10 micron.

A recent paper identified a GPI-anchored inhibitor of MMP’s called Reck, which was involved in DRG formation [12]. The authors demonstrated that *reck* and *crestin* co-localize between 22 hpf and 30 hpf, and we show co-localization here at 26 hpf (Figure 4F). We next investigated whether *reck* and *mmp17b* are co-expressed spatially and temporally during embryonic development. We performed FISH for *reck* and *mmp17b* on 24 hpf fixed embryos, and imaged double-labelled embryos using confocal microscopy. *Reck* and *mmp17b* transcripts are not detected on the same cells (Figure 4E). Triple staining for vessels (Flk-GFP), *reck* and *mmp17b* (Figure 4G & 4H), showed that *mmp17b* expressing migrating NC cells in close apposition to *reck* expressing cells in the mid-trunk region (Figure 4I). *Reck* ^+^ cells appear to delimit the position of the *crestin* ^+^ /*mmp17b* ^+^ double positive cells in the trunk. We also performed immunostaining for endogenous MMP17 (Figure 4J, white asterisk) and RECK (Figure 4K) proteins in HeLa cells, and observed that MMP17 and RECK co-localize in the same cellular compartment (Figure 4L, merge). These RNA and protein data suggest that RECK and MMP17 can interact by expression within the same cell or adjacent cells.

**Figure 5:**
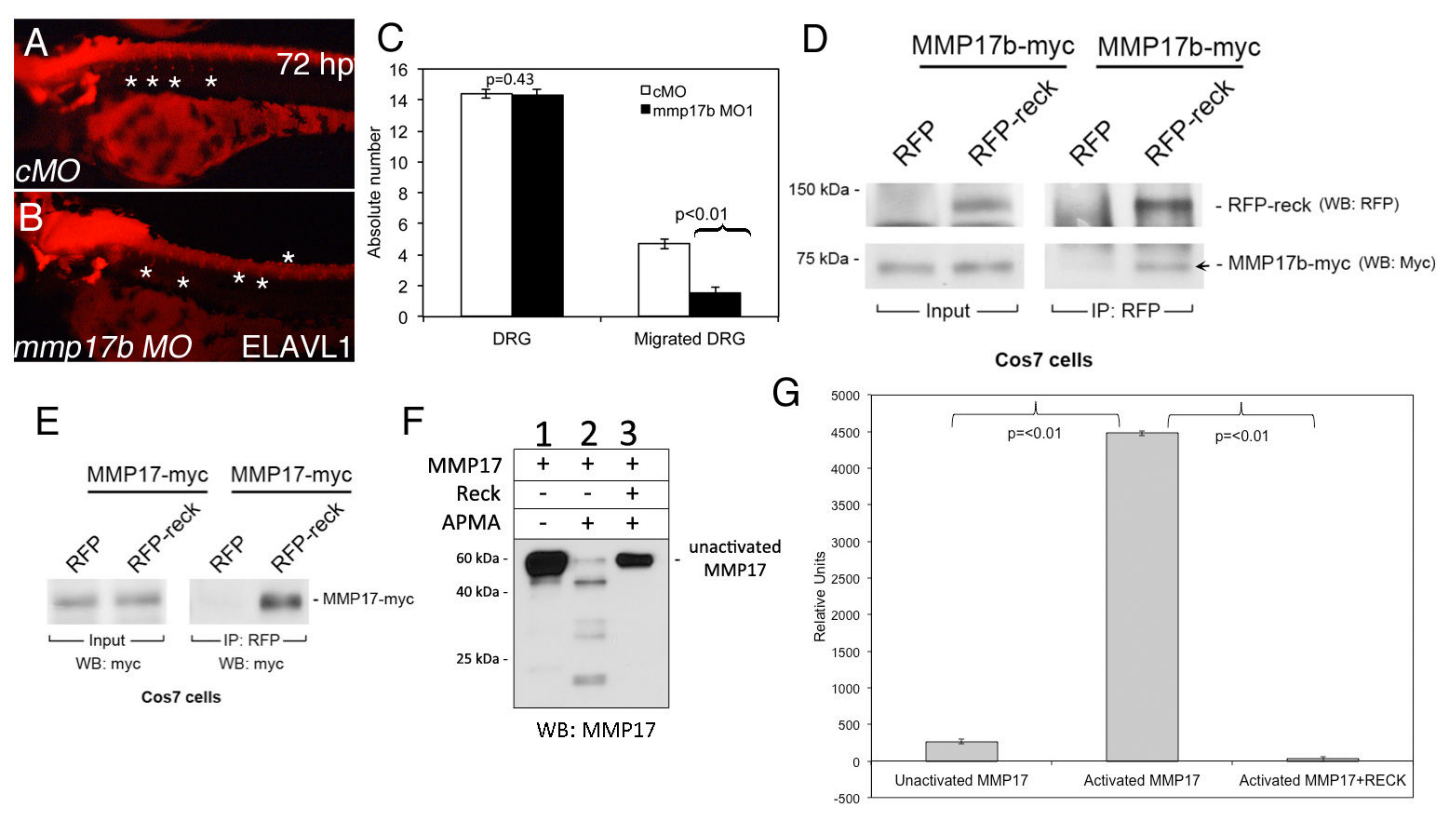
RECK blocks MMP17 activity *in vitro*. Elavl1 staining of control MO (cMO) (A) or *mmp17b* MO1 (B) injected 72 hpf embryos. Asterisks show the location of DRGs (dorsal root ganglia). *Mmp17b* knockdown embryos lack proper DRG development compared to controls. Quantitation of number of DRGs and migrated DRGs are indicated in panel C. Panel D is immunoprecipitation of myc-Mmp17b and RFP-Reck in Cos7 cells. Left panels indicate input of the two proteins in the sample, and right panels are RFP antibody immunoprecipitated samples followed by western blot. Arrow shows the pull down Mmp17b protein. Panel E is immunoprecipitation of myc-MMP17 and RFP-Reck in Cos7 cells. Left panel indicates input of MMP17 proteins in the lysate and the right panels indicate RFP antibody immunoprecipitated samples followed by western for myc epitope. Panel F is western blot of myc-MMP17 unactivated and activated with 4-aminophenylmercuric acetate (APMA) using MMP17 antibody. Activated MMP lanes show multiple bands, which are absent in samples co-incubated with RECK protein. Panel G is FRET-based fluorescence readout for MMP activity using the samples run on western blots in panel F.

Zebrafish mutant for Reck, *sensory deprived* (*sdp*), have shown failure of sensory neuron precursors to migrate to the position of the DRG [12]. To determine whether *mmp17b* and *reck* functionally interact we investigated DRG formation in *mmp17b* morphant embryos using the pan-neuronal marker elavl1. Unfortunately, knockdown of *mmp17b* in *sdp* mutant fish did not rescue the DRG phenotype, indicating that the loss of control of *mmp17b* is not the mechanism for DRG loss in this mutant. However, the *mmp17b* morphant fish embryos showed elavl1^+^ neurons located more ventrally and dorsally (Figure 5B) compared to controls (Figure 5A) suggesting defects in migration and arrangement of these neurons. Quantitation (Methods S1) in Figure 5C shows that the number of DRG’s has not altered (Figure 5C, p=0.43) but the position of the DRG’s (migrated DRG) along the axis of the embryos is clearly altered (Figure 5C, p<0.01). Next, we investigated at the biochemical level whether Mmp17b and RECK immunoprecipitated (IP) in a heterologous overexpressing Cos 7 cell system. We overexpressed tagged versions of both proteins (myc-Mmp17b, RFP-Reck) in Cos7 cells, and performed IP with RFP antibody and western blotted for myc epitope as described in Methods S1. The myc-Mmp17b protein immunoprecipitates in the RFP-Reck lane (Figure 5D, arrow), which was also observed with human MMP17 (Figure 5E). Next, we investigated whether Reck blocks MMP17 activity. We incubated pure MMP17 protein with 1 mM 4-aminophenylmercuric acetate (APMA), and performed western blot with MMP17 antibody. APMA is used to activate MMP’s in the pro(inactive)-form. The MMP17 activation event occurs *in vivo* via furin cleavage, and APMA is a chemical mimic that is used for this purpose. The activation reaction is autocatalytic and is triggered in the presence of APMA (or furin). APMA activates MMP17 as indicated by numerous smaller molecular weight bands that are noticed in APMA-treated MMP17 (Figure 5F, lane 2) compared to the untreated MMP17 (Figure 5F, lane 1). When RECK pure protein was co-incubated with APMA-activated MMP17 protein, the activation was blocked (Figure 5F, lane 3) since no lower molecular weight bands were observed. We confirmed this result by incubating the same samples in a FRET-peptide based MMP assay (Figure 5G). In this assay, the cleavage of the peptide substrate results in fluorescence. Incubation of the activated MMP17 with MMP17-specific FRET-peptide substrate results in high fluorescence values compared to unactivated MMP17 or activated MMP17 co-incubated with RECK protein. Taking the western and FRET assay data together indicates that RECK blocks the activation step of MMP17. These data collectively suggest that Reck-Mmp17b show functional interaction during DRG formation during trunk NC cell development.

## Discussion

In this study, we have characterized the function of a novel matrix metalloprotease gene, *mmp17b*, in zebrafish NC cell development. We demonstrate *mmp17b* expression in migrating NC cells, present the first demonstration of an *in vivo* function in vertebrates for a GPI-anchored MMP, and implicate functional interaction of Mmp17b and the MMP inhibitor Reck during NC cell development.

NC cell migration falls under three distinct phases (1), Directed migration resulting from contact with the ectoderm and cues from the microenvironment (2), Contact-mediated guidance facilitating homing to the target site, and (3) Contact-inhibition of movement upon entry and colonization of the target site (i.e. the ventral trunk for sympathoadrenal neurons) [4]. This behavior occurs as streams of cells that migrate directionally [24]. Although it has long been speculated that the extracellular matrix plays an active role in this process, to date the role(s) of MMPs in NC migration have remained poorly understood. Here, we implicate Mmp17b in the directed migration of NC cells in the trunk from the overlying ectoderm.

Of the 28 known human MMP’s, only two are predicted to be attached to the cell surface via a GPI anchor. Both of those, MMP17 and MMP25, have been implicated in cancer progression [25,26] but a role for these MMP’s during development has not been previously described. Here, we identify zebrafish Mmp17b whose close sequence similarity and predicted catalytic domains indicate that it is an ortholog of human MMP17. In terms of expression patterns, the only other known *mmp* to express in NC cells during development is MMP8 [27], and MMP2 [28]. At present, the GPI-anchor, the domain similarities to MMP17, and co-localization with caveolin protein argue that Mmp17b is most similar to human MMP17.

*Mmp17b* expressing NC cells at 26 hpf in the developing trunk are found in a sub-set of *crestin* ^*+*^ cells that have migrated ventrally in the anterior somites (Figure 2G), and are also found in *sox10* ^*+*^ cells (Figure 2N). This feature is more prominent in the posterior somites where the majority of *crestin* ^*+*^ cells are located in the dorsal portion of the neural tube region. Most of the *mmp17b* expressing cells are positioned further ventrally (Figure 2H) near the dorsal aorta. The *mmp17b* expression in migrating NC cells implies a functional contribution to this process, and indeed *mmp17b* LOF embryos show defects specifically in NC cell migration with little change in NC cell proliferation or apoptosis. We also examined *mmp17a*, a close ortholog of *mmp17b*, and determined that *mmp17a* expression occurred much earlier, and was not expressed in the trunk region as *mmp17b* (Figure 2K). Since 20% of the zebrafish genome is duplicated [29], it is feasible that the *mmp17a* and *mmp17b* have disparate function. To assess this we will need to perform combination knockdown analysis, which is likely to result in pre-gastrulation defect owing to the early expression of *mmp17a*. Interestingly, broad-spectrum inhibition of MMP function also leads to similar deficiencies in NC cell migration, implying that Mmp17b activity is critical for NC cell migration. Further, we did not assess for additional defects in NC migration such as precocious migration or dorsal position location of these cells. Taken together, the MMP inhibitor and *mmp17b* knockdown embryo phenotypes are similar in that the *crestin* ^*+*^ cells are misplaced, but there is no *crestin* ^*+*^ cell death (Figure S3E).

Tissue-specific metalloprotease inhibitors (TIMPs) regulate MMP activity. For MMP17, TIMP2 [30,31], TIMP3 [30], and to a lesser extent TIMP1 [30] have all been shown to inhibit MMP17 activity, and these were considered first as probable inhibitors of Mmp17b. In zebrafish TIMP1 has not been identified, TIMP3 is predicted, and two transcripts of TIMP2, *timp2a* and *timp2b* have been identified. We conducted WISH for *timp2a* and *timp2b* in 26 hpf zebrafish embryos (Figure S4D & E), and observed that *timp2a* is only expressed in the tail tip and hindbrain regions, while *timp2b* is expressed in plexus and regions adjoining or in the vasculature. We investigated the expression pattern of a GPI-anchored inhibitor of MMPs called *reck* that was recently shown to be critical for DRG formation [12]. *Reck* is co-expressed in *crestin* ^*+*^ expressing cells. At 26 hpf, *reck* (presumably *crestin* ^*+*^) expressing cells migrate ventrally from the trunk and envelops *mmp17b* expressing NC cells (Figure 4G, asterisk) in the middle of the trunk. Loss of *mmp17b* in embryos show *crestin* ^+^ cells near the dorsal region of the neural tube at the posterior end of the embryo at 26 hpf. However, loss of *reck* causes *sox10* ^+^ cells (presumably *crestin* ^+^) NC cells to increase their velocity of migration ventrally [12] implying that presence of Mmp17b is responsible for directional migration of NC cells from dorsal ectoderm to ventral regions of the embryo.

The juxtaposition of *mmp17b* expressing cells with *reck* expressing cells implies that the putative interaction of the two cells will most likely trigger signals that dictate the final location of the differentiated DRGs. At a molecular level, RECK protein is capable of blocking the activation of MMP17 *in vitro*, which in itself suggests that MMP17 is a new, previously unreported target of RECK. Further support for this hypothesis is from *mmp17b* LOF embryos data where the number of DRGs has not changed presumably because of the presence of Reck but their absolute position on the dorsal-ventral axis is affected. This suggests that Reck is necessary for specification of DRGs from NC precursors, and Mmp17b is necessary for the DRGs final position. Taking our data and those of Prendergast et al [12] together, we hypothesize that Reck is responsible for the NC cell differentiation into DRGs post migration dorsally after 30 hpf. Concomitantly or slightly earlier, *mmp17b* expressed in a dorsal NC cell population migrate ventrally until they are properly oriented with *reck* expressing cells from the ventral region. In this scenario, loss of *reck* would result in NC cells not receiving the proper cues to differentiate into DRGs resulting in loss of DRGs as observed in *sdp* mutants. Loss of *mmp17b* however, results in NC cells differentiating into DRGs because of Reck presence but they do not migrate to the proper position in the zebrafish trunk. Future studies will need to elaborate the nature of this interaction and signalling mechanisms in this process.

In terms of melanocytes that differentiate from trunk NC cells, disruption of NC cell migration in *mmp17b* LOF embryos results in no change in number of melanocytes but alters their migration to the final position, which implies that the matrix and surrounding cells does influence the final positioning of the differentiated NC cells. In addition to melanocytes, *mmp17b* morphants also showed defects in tail fin skeletal structure (Figures S4B & S4C) that is likely to be derived from NC cells [32]. This defect was also observed in MMP-inhibitor-treated embryos, and is likely to be secondary to alteration in trunk NC cell migration events. However, recent studies show that NC does not play a role in larval fin development [33]. We speculate that the tail and fin abnormalities could be due to disruption in cartilage development in this region via collagen processing, because human MMP17 is known to play a role in the activation of a disintegrin and metalloproteinase with thrombospondin-like motif-4 (ADAMTS-4) [34,35] during cartilage breakdown. Other possibilities include regulating the levels of growth factor sequestered in the matrix. For example, MMP17 could behave like MMP9 in that it facilitates the availability of sequestered VEGF in the matrix [36].

In summary, this study is the first to unravel the molecular role for Mmp17b in vertebrate development, and suggests a functional role for this molecule and its cognate TIMP Reck during NC cell development.

## Materials and Methods

### Ethics Statement

All zebrafish studies were performed according to Medical College of Wisconsin animal protocol guidelines under protocol AUA320. The MCW’s IACUC committee approved studies conducted here.

### Zebrafish Strain and Transgenic Lines

Fish of the TübingenAB (TuAB) wild-type strain and transgenic kinase insert domain receptor enhanced green fluorescent protein (*Tg*[*kdr*:EGFP]) [37] line were obtained from ZIRC (Eugene, OR). The *sensory deprived* (*sdp*) mutant fish were obtained from the laboratory of Dr. David Raible at the University of Washington. Transgenic 7.2 kb *sox10*-promoter-driven membrane-localized red fluorescent protein (*Tg*[*sox10*:mRFP]vu234) [18] fish were a kind gift of the laboratory of Dr. Bruce Appel at the University of Colorado-Denver.

### Morpholino Injections

Gene Tools, Inc. designed splice MOs targeting the exon-intron junction of exons 3 (*mmp17b* MO1) and 4 (*mmp17b* MO2) of the *mmp17b* gene (Figure S2B). All primers and morpholino sequences used in this study are provided in Table 1. Both gene-specific and control MOs were reconstituted to a final concentration of 2 mM using water. Microinjection was performed with two nanoliters (nL) injected into each embryo at 1-2 cell stage. Doses of MO were empirically determined and were 15 ng - Control MO (cMO), 12.5 ng – *mmp17b* MO2, 7.5 ng – *mmp17b* MO1. Concentration of MO was chosen that showed the most minimal gastrulation delay (i.e., most normal looking), and yet showed defects in marker staining. Each injection experiment was repeated three independent times with approximately 40 embryos/condition each time. For MO targeting, RT-PCR was performed with primers located in the adjacent exons as shown in Figure S2. To determine the consequence of MO targeting, the alternate bands identified in the MO-targeted lanes were cloned into the CloneSmart^®^ HCKan vector using the CloneSmart^®^ Blunt Cloning Kit (Lucigen^®^) and sequenced.

### Whole Mount *In Situ* Hybridization (WISH)

Zebrafish *mmp17b, timp2a*, and *timp2b* cDNA was generated from total zebrafish 24 hpf RNA using RT-PCR. The RT reaction conditions included: 1.5 µg of RNA, 1 µL of 50 µM oligo dT_20_ (Invitrogen^™^) 10 mM dNTP, in a 15 µL reaction. Standard RT-PCR protocols were carried out with a slight modification in that the Phusion^®^ DNA Polymerase (Finnzymes) was used in the PCR reaction to amplify the full-length *mmp17b, timp2a* or *timp2b* fragment. The amplified PCR fragment was used as a template in future PCR amplifications for probe generations. Initially, T3 and T7 promoter sequences were designed into the forward and reverse primers for generating template that were used in subsequent *in vitro* transcription to produce digoxigenin-labeled RNA-probes. Subsequently, we cloned the full-length *mmp17b* fragment into pcr4-TOPO vector (Life Technologies), linearized the plasmid with NotI enzyme, and the T3 sequences in the plasmid were used for probe generation as described previously [38]. The WISH procedure was performed as described before [38]. Two-color fluorescent in-situ-hybridization (FISH) was performed with the following modifications: Instead of single riboprobe incubation, embryos were incubated with pairs of digoxygenin (DIG)- and dinitrophenol (DNP)-labeled riboprobes. Labeling of riboprobes was done by *in vitro* transcription from PCR-generated probe templates. We used a DIG RNA labeling mix (Roche) to produce DIG-labeled riboprobes, and DNP-labeled riboprobes were produced using a mix of ribonucleotides and DNP-11-UTP (PerkinElmer). After hybridization, stringent washes in PBST (PBS, 0.1% Tween-20) and blocking of embryos in block solution (1% BSA and 1% DMSO in PBST, 2 h at room temperature [RT]) were done. RNA-DIG probe hybrids of the first transcript were detected by overnight incubation at 4°C with anti-DIG Fab fragments conjugated to horseradish peroxidase (Roche, 1:1000 dilution) in block solution. Fluorescent signal was produced using Cy3, fluorescein or Cy5 tyramide signal amplification (TSA) systems (PerkinElmer, 1:100 dilution, 30-40 min incubation at room temperature) as the first color. Following washes in PBST, quenching of peroxidase activity (at least 1 hour incubation in 1.5% H_2_O_2_ in PBST), and further extensive PBST washes and another blocking step, embryos were incubated with anti-DNP antibody (PerkinElmer, 1:500 dilution in block solution) overnight at 4°C. Then the second fluorescent signal was developed using a different tyramide substrate than in the first round. Embryos were washed thoroughly in PBST and ready for imaging. Details on the double staining procedure are available upon request. The embryos were mounted using PolyMount solution (Polysciences) and allowed to dry overnight in darkness. The embryos were imaged the next day using a Zeiss LSM 510 confocal microscope. The images were analyzed using Volocity software (PerkinElmer).

In some cases, we performed immunofluorescence (IF) after double FISH to yield three-color images. The embryos were incubated for 2 h in block solution at RT followed by incubation with primary antibody (for GFP [Torrey Pines Laboratories] rabbit anti-GFP 1:1000; for elavl1 [Invitrogen A21271] 1:500 dilution) over night at 4°C. The following day embryos were washed five times in PBST+1% DMSO for 5 min each, and re-blocked as above for 2 h at RT. After blocking, the embryos were incubated for 2 h in secondary antibody at RT (for GFP goat anti-rabbit Alexa 488 [Invitrogen A11008, 1:1000]; for elavl1 goat anti-mouse Alexa 568 [Invitrogen A11004, 1:1000]), followed by five washes with PBST+1% DMSO for 5 min each. Similarly, after single *mmp17b* FISH using fluorescein-tyramide (PerkinElmer), embryos were washed, re-blocked, and incubated with anti-RFP primary antibody (Invitrogen R10367, 1:1000) for sox10 detection. Secondary antibody was anti-rabbit Alexa 647 (Invitrogen A21244, 1:1000). Antibodies against znp-1 (1:1000 dilution) and zn-1 (1:500 dilution) were mouse hybridoma supernatants from ZIRC (Eugene, OR) developed with anti-mouse-Alexa488 (Jackson ImmunoResearch Laboratories, 1:500 dilution). Since co-detection of *mmp17b* involved DNP-labeled riboprobes recognized by mouse anti-DNP-HRP, detection of znp-1/zn-1 and washing off unbound anti-mouse-Alexa488 secondary antibody was done before introduction of mouse anti-DNP-HRP and Cy5-tyramide to localize *mmp17b* transcripts. All antibody incubations occurred in block solution overnight at 4°C. Following extensive washes in PBST, the embryos were mounted as described above and imaged using either the Zeiss confocal microscope (FISH, GFP, RFP IFs) or Zeiss Observer Z1 microscope (elavl1 IF).

### Chemical Inhibitor Analysis

Two broad-spectrum inhibitors Marimastat and ONO-4817 (Tocris Bioscience) were reconstituted to a concentration of 100 mM in 100% DMSO. The drug was diluted in E3 embryo water to produce working stocks. Ranges of concentration (0.5 mM, 50 µM, 5 µM, and 0.5 µM) were titrated for both drugs to determine a dose that yielded a phenotype without grossly disrupting the development of the embryo similar to the MO protocol. Total DMSO concentration was kept at 0.5%. Tg(*kdr*:EGFP) zebrafish embryos were punctured at 10 hpf with forceps to facilitate drug diffusion into the embryo. Ten embryos (10 hpf stage) were placed per well in a 24-well plate, and treated with 0.5 mL of either a drug or a DMSO control until the embryos reached 24 hpf stage at 28.5°C. The embryos were then dechorionated and analyzed for abnormalities similar to the MO injection described earlier. Photography and imaging was also performed as described above. The concentration of drug that was optimal for these studies was 0.5 mM. The experiment was repeated twice, and all abnormalities were quantitated, documented, and graphed. WISH for *crestin* and *mmp17b* was conducted on the drug treated embryos as described previously.

### MMP17 Activation and FRET-peptide MMP Assay

Human recombinant MMP17 (R&D Systems) was first diluted to 100 µg/mL in Activation Buffer (1M Tris, 1M CaCl_2_, 5M NaCl, 30% Brij-35, 0.1M ZnCl_2_, pH 7.5) and was then activated by incubating in 1 mM APMA (4-Aminophenylmercuric acetate, Sigma-Aldrich) for 2 h at room temperature. After activation, the MMP17 protein was diluted 1:50 in Assay Buffer (1M Tris and 1M CaCl_2_, pH 7.5) and 50 µl was plated in a black 96-well plate. MMP17 fluorogenic substrate (Mca-PLAQAV-Dpa-RSSSR-NH_2_-Fluorogenic Peptide Substrate III, R&D Systems) was diluted 1:200 in Assay Buffer and 50 µl was added to either experimental sample or 50 µl Assay Buffer for substrate control. After mixing the substrate with the samples, the plate was covered and protected from light and allowed to incubate at room temperature for 1 h. The plate was then read in a Molecular Devices Gemini EM fluorescent plate reader on the EndPoint setting. Each sample was read for 10 seconds at Excitation 320 nm and Emission 405 nm. This assay was performed twice, and gave reproducible results.

## Supporting Information

Figure S1 **Bioinformatic analysis of Mmp17b.** Panel A depicts amino acid alignment of Mmp17b with various related MMP proteins. Red colors indicate conserved amino acids and blue colors indicate less conserved regions. Panel B shows how related Mmp17b is to other related MMP proteins by amino acid sequence phylogeny.
(TIF)

Figure S2 ***mmp17b* expression, knockdown efficacy and role in early development.** A shows three color staining of the trunk of a 26 hpf embryo for motor neurons. Upper left panel is *mmp17b* in purple, lower left panel is znp-1/zn-1 motor neuron staining in green, lower right panel is Flk-GFP staining endothelial cells in red, and the upper right panel is a merge. The upper right panel shows co-localization of *mmp17b* and znp-1/zn-1 staining. B shows cartoon illustrating where the *mmp17b* morpholinos (MO) were targeted. MO1 and MO2 targeted the exon-intron boundary of exons 3 and 4 respectively. Blue arrows indicate start of primers used to confirm efficacy of MO1. Red arrows indicate start of primers used to confirm efficacy of MO2. C shows efficacy for MO1 and MO2 demonstrated via RT-PCR. Primers illustrated in panel B were used to amplify *mmp17b* fragments. In both MO1 and MO2, the *mmp17b* amplicon was larger than the control MO injected sample. In MO1, there appeared to be a major higher band along with a minor band that was consistent with the normal *mmp17b* band. In MO2, there was a major band much higher than control and a minor band consistent with the higher band observed in the MO1 sample. Asterisks indicate the aberrant transcripts. β-actin was used as a loading control. D shows knockdown of *mmp17b* does not affect early development. When *etv-2* (upper and lower left panels) and *myoD* (upper and lower right panels) are probed for in 14 hpf *mmp17b* MO1 injected embryos (lower left and right panels), there is no difference observed compared to control MO injected embryos (upper left and right panels).
(TIF)

Figure S3 **Proliferation and cell death in *mmp17b* knockdown embryos.** A and B are structures of MMP inhibitors Marimastat and ONO-4817 used in this study. C-E are TUNEL assay staining performed on uninjected (UI) (C) and *mmp17b* MO1 (E) injected 26 hpf embryos. A 26 hpf control MO injected embryo treated with 6 mM H_2_O_2_ (D) was included as a positive control. F-H are phosphohistone H3 (pH3) staining performed on control MO (F), *mmp17b* MO1 G), and *mmp17b* MO2 (H) injected 26 hpf embryos. No difference was observed in *mmp17b* KD embryos compared to controls.
(TIF)

Figure S4 **Cartilage and vascular defects observed in *mmp17b* knockdown fish.** A-B are bright field images of control MO (A) and *mmp17b* MO1-injected (B) 48 hpf embryos. Panel B illustrates the cupped-fin phenotype seen in *mmp17b* MO1-injected embryos compared to controls. This defect was quantitated in panel C that shows *mmp17b* MO1-injected embryos have a statistically significant increase in the cupped-fin defect compared to controls. Panels D and E show *timp2a* and *timp2b* 26 hpf ISH embryos (head is to the left). Yellow arrow in D shows plexus region and the black arrow indicates regions adjoining the vasculature.
(TIF)

Methods S1 **Supporting Methods.**
(DOCX)
