# Supplementary figures and images for: Mmp17b Is Essential for Proper Neural Crest Cell Migration In Vivo
Source: PLoS One. 2013 Oct 1;8(10):e76484. doi: 10.1371/journal.pone.0076484 (PMC3788140; doi:10.1371/journal.pone.0076484)

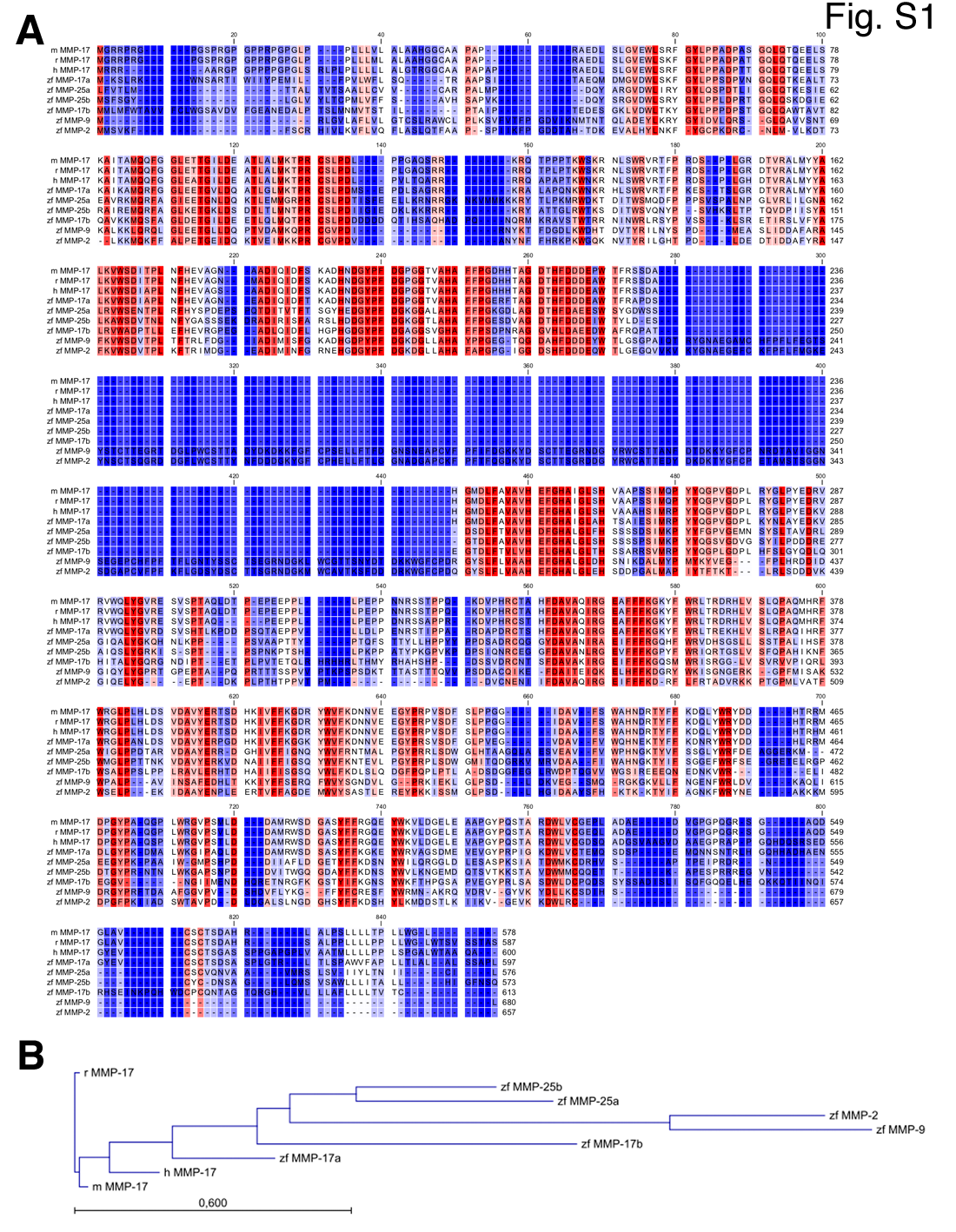

Supplement: Figure S1 — Bioinformatic analysis of Mmp17b. Panel A depicts amino acid alignment of Mmp17b with various related MMP proteins. Red colors indicate conserved amino acids and blue colors indicate less conserved regions. Panel B shows how related Mmp17b is to other related MMP proteins by amino acid sequence phylogeny. (TIF) [file pone.0076484.s001.tif]

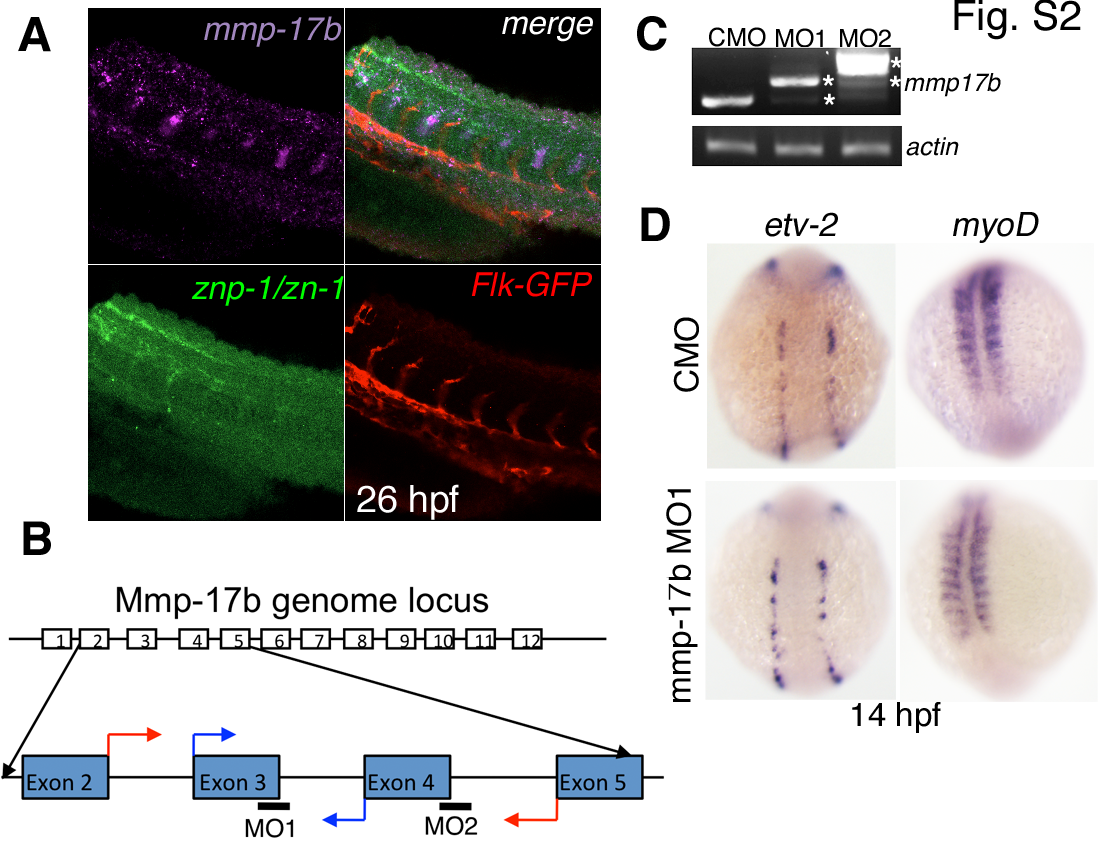

Supplement: Figure S2 — mmp17b expression, knockdown efficacy and role in early development. A shows three color staining of the trunk of a 26 hpf embryo for motor neurons. Upper left panel is mmp17b in purple, lower left panel is znp-1/zn-1 motor neuron staining in green, lower right panel is Flk-GFP staining endothelial cells in red, and the upper right panel is a merge. The upper right panel shows co-localization of mmp17b and znp-1/zn-1 staining. B shows cartoon illustrating where the mmp17b morpholinos (MO) were targeted. MO1 and MO2 targeted the exon-intron boundary of exons 3 and 4 respectively. Blue arrows indicate start of primers used to confirm efficacy of MO1. Red arrows indicate start of primers used to confirm efficacy of MO2. C shows efficacy for MO1 and MO2 demonstrated via RT-PCR. Primers illustrated in panel B were used to amplify mmp17b fragments. In both MO1 and MO2, the mmp17b amplicon was larger than the control MO injected sample. In MO1, there appeared to be a major higher band along with a minor band that was consistent with the normal mmp17b band. In MO2, there was a major band much higher than control and a minor band consistent with the higher band observed in the MO1 sample. Asterisks indicate the aberrant transcripts. β-actin was used as a loading control. D shows knockdown of mmp17b does not affect early development. When etv-2 (upper and lower left panels) and myoD (upper and lower right panels) are probed for in 14 hpf mmp17b MO1 injected embryos (lower left and right panels), there is no difference observed compared to control MO injected embryos (upper left and right panels). (TIF) [file pone.0076484.s002.tif]

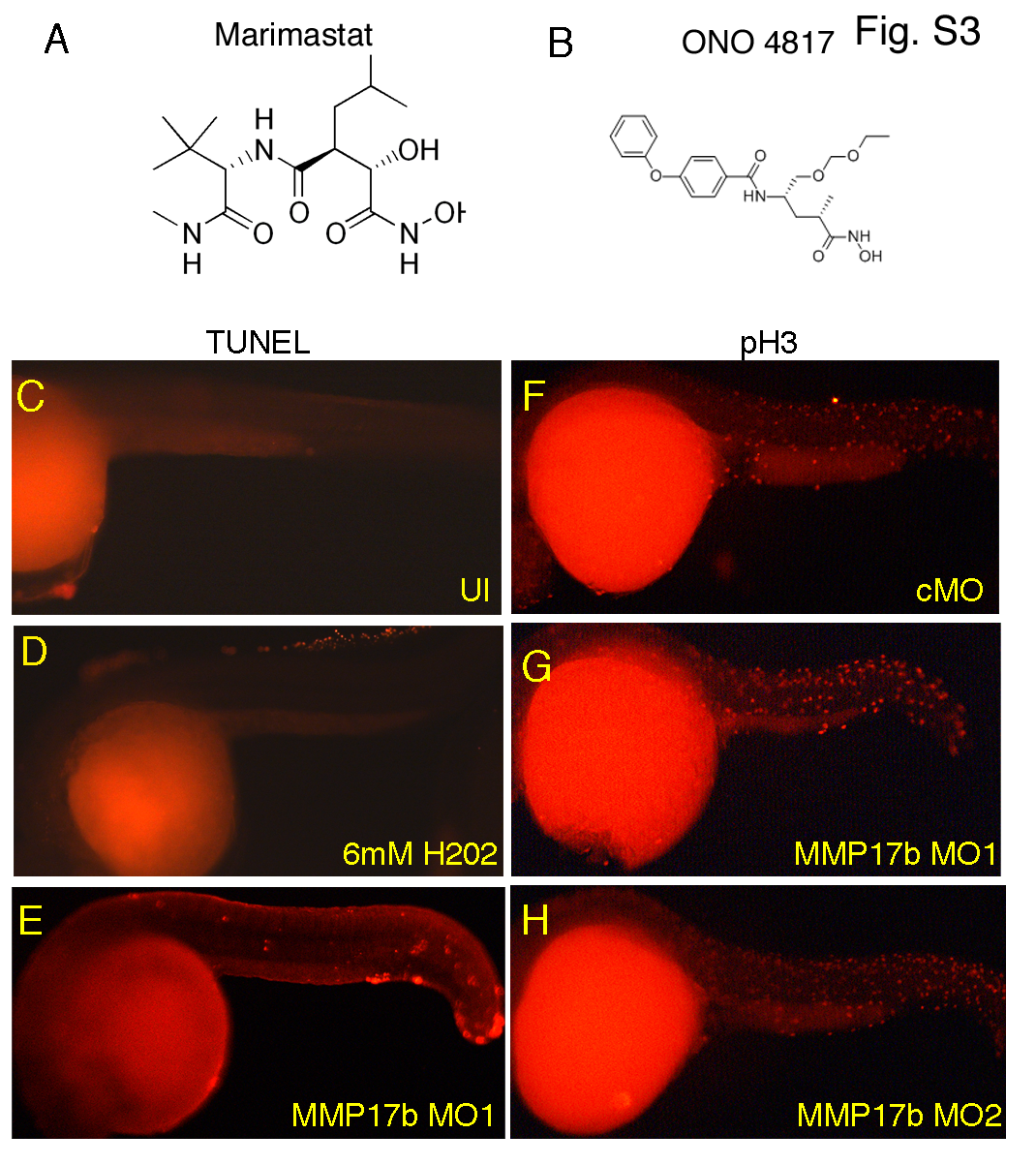

Supplement: Figure S3 — Proliferation and cell death in mmp17b knockdown embryos. A and B are structures of MMP inhibitors Marimastat and ONO-4817 used in this study. C-E are TUNEL assay staining performed on uninjected (UI) (C) and mmp17b MO1 (E) injected 26 hpf embryos. A 26 hpf control MO injected embryo treated with 6 mM H2O2 (D) was included as a positive control. F-H are phosphohistone H3 (pH3) staining performed on control MO (F), mmp17b MO1 G), and mmp17b MO2 (H) injected 26 hpf embryos. No difference was observed in mmp17b KD embryos compared to controls. (TIF) [file pone.0076484.s003.tif]

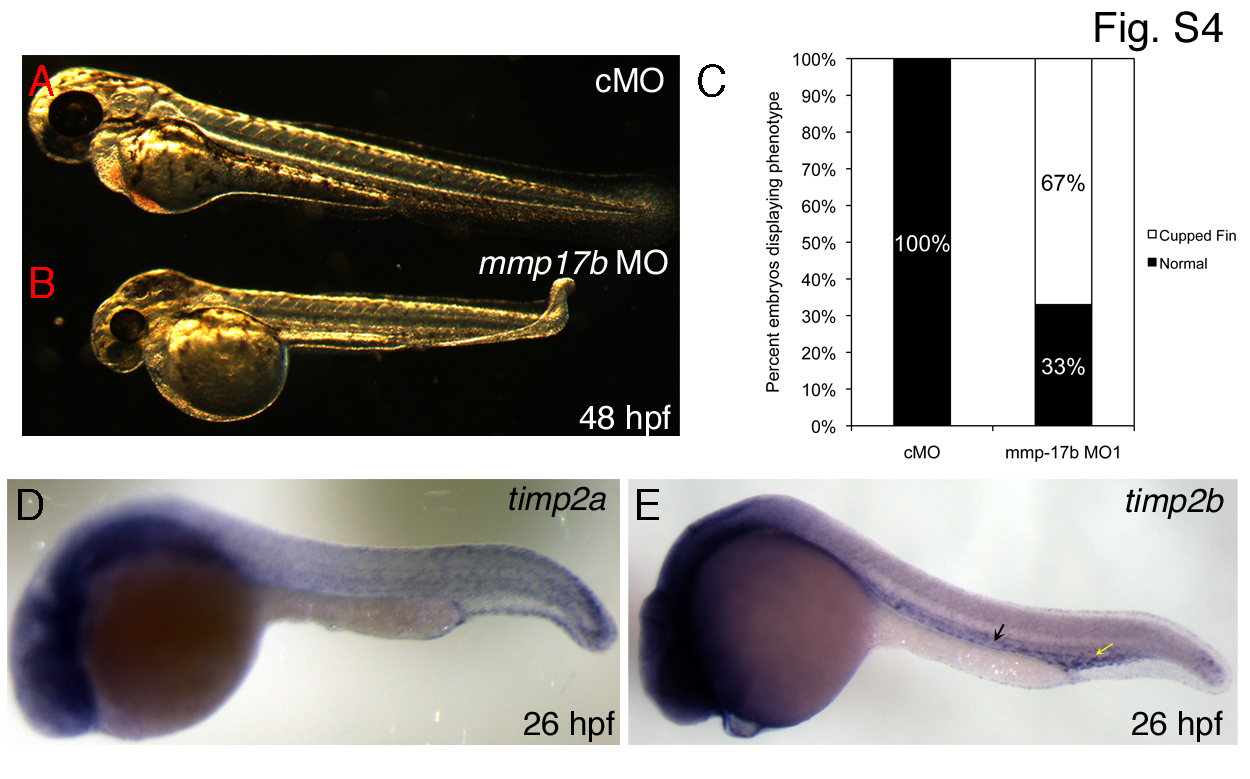

Supplement: Figure S4 — Cartilage and vascular defects observed in mmp17b knockdown fish. A-B are bright field images of control MO (A) and mmp17b MO1-injected (B) 48 hpf embryos. Panel B illustrates the cupped-fin phenotype seen in mmp17b MO1-injected embryos compared to controls. This defect was quantitated in panel C that shows mmp17b MO1-injected embryos have a statistically significant increase in the cupped-fin defect compared to controls. Panels D and E show timp2a and timp2b 26 hpf ISH embryos (head is to the left). Yellow arrow in D shows plexus region and the black arrow indicates regions adjoining the vasculature. (TIF) [file pone.0076484.s004.tif]
