# Supplementary material for: Mmp17b Is Essential for Proper Neural Crest Cell Migration In Vivo
Source: PLoS One. 2013 Oct 1;8(10):e76484. doi: 10.1371/journal.pone.0076484 (PMC3788140; doi:10.1371/journal.pone.0076484)
Supplement: Methods S1 — Supporting Methods. (DOCX) [file pone.0076484.s005.docx]

**Supporting Information**

**SUPPORTING METHODS**

**Bioinformatic analysis.** We searched the zebrafish information network (ZFIN) database using search terms artery, vein, and vascular, and identified gene entry sb:eu434. The sb:eu434 sequence retrieved by the accession number Mmp17b is located in the predicted transcript *GENSCAN00000013789,* chromosome 14 on Ensemble Zv8. The gene was also predicted by Gnomon as the transcript *hmm162364* on Zv7 contig Dr14_WGA1412_3. Alignment of the human, rat, and mouse MMP17, and the zebrafish Mmp17a, Mmp17b, Mmp25a, Mmp25b, Mmp2, and Mmp9 were done using CLC Free Workbench 3 program. Domain analysis was conducted using the deposited ZFIN sequence using Simple Modular Architecture Research Tool (SMART) website and a GPI anchor site was also predicted at residue 592 using the big-PI Predictor website. Protein tree analysis was done using the same sequences as above and utilized CLC Free Workbench 3 program.

**Cloning MMP17b and Expression.** The full-length zebrafish *mmp17b* cDNA was PCR amplified from a previous clone in the lab using the Expand High Fidelity PCR system as per the manufacturer’s instruction (Roche Applied science; USA). The PCR product (1.845 kb) was Kpn I and Not I digested and sub-cloned into pcDNA3.1(+)- myc-his-C vector. Eukaryotic expression of Mmp17b-myc-his fusion protein was validated by transient transfection of 8 μg of DNA into HEK293T cells using Lipofectamine 2000 (Life technologies, USA) for 48 h, which were compared to the either 2 μg of pcDNA3.1(+)-myc-his-C without insert as a negative control or 2 μg of IRSP53-myc-his fusion protein as a positive control by western blotting. Cell lysates were prepared using RIPA buffer supplemented with protease and phosphatase inhibitor (Roche, USA), incubated for 20 min for complete lysis. A supernatant (S) and pellet (P) were collected by centrifugation at full speed using a tabletop centrifuge. The same procedure as above was used for Cos7 cell transfection as well except the negative control was untransfected Cos7 cell lysates, and the positive control was 4 μg of human MMP17 and human MMP25 (Origene, USA).

**Phenotype Determination and Quantitation.** The embryos were viewed using a Leica MZ16FA upright microscope equipped with a QImaging Retiga EXi camera to observe whether physical abnormalities are seen in injected fish. Defective patterning was defined as either disrupted crestin staining or lack of crestin staining below the midline of the trunk of the fish. Normal staining was defined as continuous crestin staining from the dorsal to just before the ventral end of the embryo with the staining getting progressively thinner as it moved from dorsal to ventral. Total number of embryos exhibiting abnormal crestin staining was determined for control MO injected, *mmp17b* MO1, and MO2 injected embryos and the results were graphed. Embryo pictures, both whole mount and the trunk regions, were captured at 26 hpf as described previously. Fluorescent images were obtained using the 1X objective and a 488 nm filter. Both bright field and fluorescent images of the whole embryo were taken and comparable fluorescent images of the trunk region were also captured for control and *mmp17b* MO injected fish.

To quantify melanocytes, embryos were injected with control MO or *mmp17b* MO1 and allowed to develop until 48 hpf. The embryos were imaged using a Leica MZ16FA upright microscope equipped with a QImaging Retiga EXi camera. Images were taken from a dorsal view of the fish and the number of melanocytes was quantitated in two regions between the first and seventh somite. The first region was along the midline of the fish and the second region was on either side of the midline. The total number in each region was quantitated for 10 fish from each condition and compared to control MO injected fish. The numbers were averaged and graphed with standard error bars.

Dorsal Root Ganglion (DRG) were quantitated on control MO injected and *mmp17b* MO injected embryos at 48 hpf stained with elavl1 antibody. The embryos were imaged from the dorsal view and number of DRGs was counted and length of DRGs extending from midline was measured. The results of ten representative embryos were then graphed with standard error bars.

**Cell Death and Proliferation Analysis.** To determine apoptosis *in vivo*, a Terminal Uridine Nick-End Labeling (TUNEL) assay was conducted as described previously [[1](#_ENREF_1)]. Images were obtained using a Leica MZ16FA upright microscope equipped with a QImaging Retiga EXi camera and a Cy3 filter. For cell proliferation, phospho-histone H3 staining was conducted as described previously [[2](#_ENREF_2)]. Anti-pH3 primary was used at a 1:500 dilution (Cell Signalling), and a donkey anti-rabbit Cy3-AffiniPure secondary antibody was used at a 1:1000 dilution (Jackson Immuno).

**Cell Culture and immunofluorescence microscopy.** HEK293T, Cos7 and HeLa cells were used in this study, and were maintained in the recommended media prescribed by ATCC. Cells were grown under standard cell culture procedures such as humidified conditions in 5% CO_2_ and 37°C. For IF, HEK293T cells were seeded on glass coverslips at a density of approximately 50%. The following day, a set of four coverslips were transiently transfected with human- *MMP17*-myc tag (OriGene, USA) and *mmp17b*-myc-HIS tag cDNA plasmids (3 µg each) respectively using Lipofectamin-2000 (Life technologies, USA). Post 12 h of transfection cells were cultured in regular media for additional 30 h. Subsequently, cells were washed in warm PBS and fixed using 4% Para-Formaldehyde (PFA) for 10 min and followed by permeabilization for 5 min in 0.5% Triton X-100. For immunofluorescence, primary antibodies (human MMP17 from OriGene; USA raised in rabbit and human Caveolin antibody from BD transduction laboratories, USA raised in mouse) were diluted in PBST buffer (1:100), incubated for one hour followed by a brief 5 min wash with PBST three times. The secondary antibody conjugates to detect the primary antibodies were goat anti mouse Alexa-fluorophore-568 and goat anti rabbit Alexa-fluorophore-488, diluted in PBST (1:1000), along with DAPI were used for about 1 h. The coverslips were washed and mounted on to glass slides for microscopy. Representative images were acquired using Zeiss observer.Z1 imaging system controlled by the Axiovision Rel 4.8 software. To detect the localization of RECK (mAb (clone 198212) from R & D Systems) with respect to MMP17, HeLa cells were cultured to sub-confluence and processed as described above.

**Western Blotting and Immunoprecipitation (IP):** Western blotting was performed with SDS-PAGE electrophoresis system as described previously [[3](#_ENREF_3)]. Primary antibodies include myc tag antibody (1:1000) (Cell Signaling Technologies, USA) or human MMP17 antibody (1:1000) (Origene, USA) and secondary antibodies include HRP conjugated anti-mouse or anti-rabbit antibody (Cell Signaling Technologies, USA) at 1:10000. The membranes were developed with Pierce ECL Western Blotting Substrate kit per manufacturer’s instructions. Dr. David Raible at the University of Washington, Seattle provided the RFP-Reck constructs used in the IP experiment. For IP, Cos7 cells were co-transfected, with the indicated cDNAs. After 24 h, cells were lysed in buffer containing 0.5% NP-40, 5 mM EGTA, 120 mM NaCl, 50 mM Tris-HCl (pH 7.4), 1 mM DTT, and protease and phosphatase inhibitors. The lysates were then cleared at 2500 x *g* for 10 min at 4°C. The resulting supernatants were immunoprecipitated with mouse anti-RFP (Invitrogen, R10367) and a 50/50 mix of protein A/G agarose beads. Immunoprecipitates were washed 3x with dilution buffer (10 mM Tris, 130 mM NaCl, 0.05% Triton X-100, 0.1% BSA, protease inhibitors) and once with 50 mM Tris (pH 7.9). Immunoprecipitates and total cell lysates were subjected to ECL-Western blotting with anti-RFP and anti-Myc antibodies (1:125) (Cell Signaling, 9B11).

**SUPPORTING REFERENCES**

1. Chun CZ, Kaur S, Samant GV, Wang L, Pramanik K, et al. (2009) Snrk-1 is involved in multiple steps of angioblast development and acts via notch signaling pathway in artery-vein specification in vertebrates. Blood 113: 1192-1199.

2. Chun CZ, Remadevi I, Schupp MO, Samant GV, Pramanik K, et al. (2011) Fli+ etsrp+ hemato-vascular progenitor cells proliferate at the lateral plate mesoderm during vasculogenesis in zebrafish. PLoS One 6: e14732.

3. Kaur S, Castellone MD, Bedell VM, Konar M, Gutkind JS, et al. (2006) Robo4 signaling in endothelial cells implies attraction guidance mechanisms. J Biol Chem 281: 11347-11356.
